# Amino Acid Metabolism in Lupus

**DOI:** 10.3389/fimmu.2021.623844

**Published:** 2021-02-22

**Authors:** Michihito Kono, Nobuya Yoshida, George C. Tsokos

**Affiliations:** ^1^ Department of Rheumatology, Endocrinology and Nephrology, Faculty of Medicine, Hokkaido University, Sapporo, Japan; ^2^ Department of Medicine, Beth Israel Deaconess Medical Center, Harvard Medical School, Boston, MA, United States

**Keywords:** cell metabolism, amino acid, T cell, systemic lupus erythematosus, amino acid transporters

## Abstract

T cell metabolism is central to cell proliferation, survival, differentiation, and aberrations have been linked to the pathophysiology of systemic autoimmune diseases. Besides glycolysis and fatty acid oxidation/synthesis, amino acid metabolism is also crucial in T cell metabolism. It appears that each T cell subset favors a unique metabolic process and that metabolic reprogramming changes cell fate. Here, we review the mechanisms whereby amino acid transport and metabolism affects T cell activation, differentiation and function in T cells in the prototype systemic autoimmune disease systemic lupus erythematosus. New insights in amino acid handling by T cells should guide approaches to correct T cell abnormalities and disease pathology.

## Introduction

Systemic lupus erythematosus (SLE) is a chronic autoimmune disease characterized by autoantibody production, immune complex deposition, tissue inflammation and damage of multiple organs (1). SLE can affect practically all organs, including skin, kidney, and central nerve system (2–4). The etiology of SLE is multifactorial and includes contributions from genetic, environmental, hormonal and epigenetic factors (2). These factors, acting serially or simultaneously, lead to generalize breakdown of tolerance to self-antigens, which results in autoantibody production and tissue inflammation (5). T cells have a vital role in the pathogenesis of SLE. Many subsets of T cells, especially Th1, Th17, regulatory T (Treg) cells, and double-negative (CD4^-^CD8^-^) T cells, are involved through distinct mechanisms in the development of organ inflammation in SLE (6). Since helper T cells can activate B cells to secrete antibodies, which are also involved in the lupus pathogenesis, T cells have earned claim as main therapeutic targets in patients with SLE (7).

Recent studies have shown that the differentiation and function of each T cell subset is controlled by intracellular metabolic processes (8–10). Cell metabolism operates mainly through glycolysis, fatty acid oxidation and amino acid metabolism including glutaminolysis (8–11). Amino acids are classified as essential (leucine, isoleucine, lysine, histidine, valine, threonine, phenylalanine, tryptophan, and methionine), conditionally essential (glutamine, arginine, cysteine, glycine, proline, and tyrosine), or non-essential (alanine, glutamate, serine, asparagine, and aspartate) (12). Essential amino acids cannot be synthesized within the body and must be supplied through dietary intake. Amino acid metabolism is used in many processes that are involved in cell proliferation, growth and cell function. Furthermore, amino acids are also critical for the biosynthesis of nucleotides (13). It has been documented that some amino acids such as leucine, methionine, glutamine, arginine, and alanine, are more essential than other amino acids during T cell activation and expansion or in determining distinct T cell fates (14, 15). The importance of glycolysis, and fatty acid oxidation/synthesis in lupus T cells has been extensively reviewed elsewhere (8–10, 16–18). Here we summarize amino acid metabolism in mice and people with SLE with a focus on T cells.

## Amino Acid Transporters

**Figure 1:**
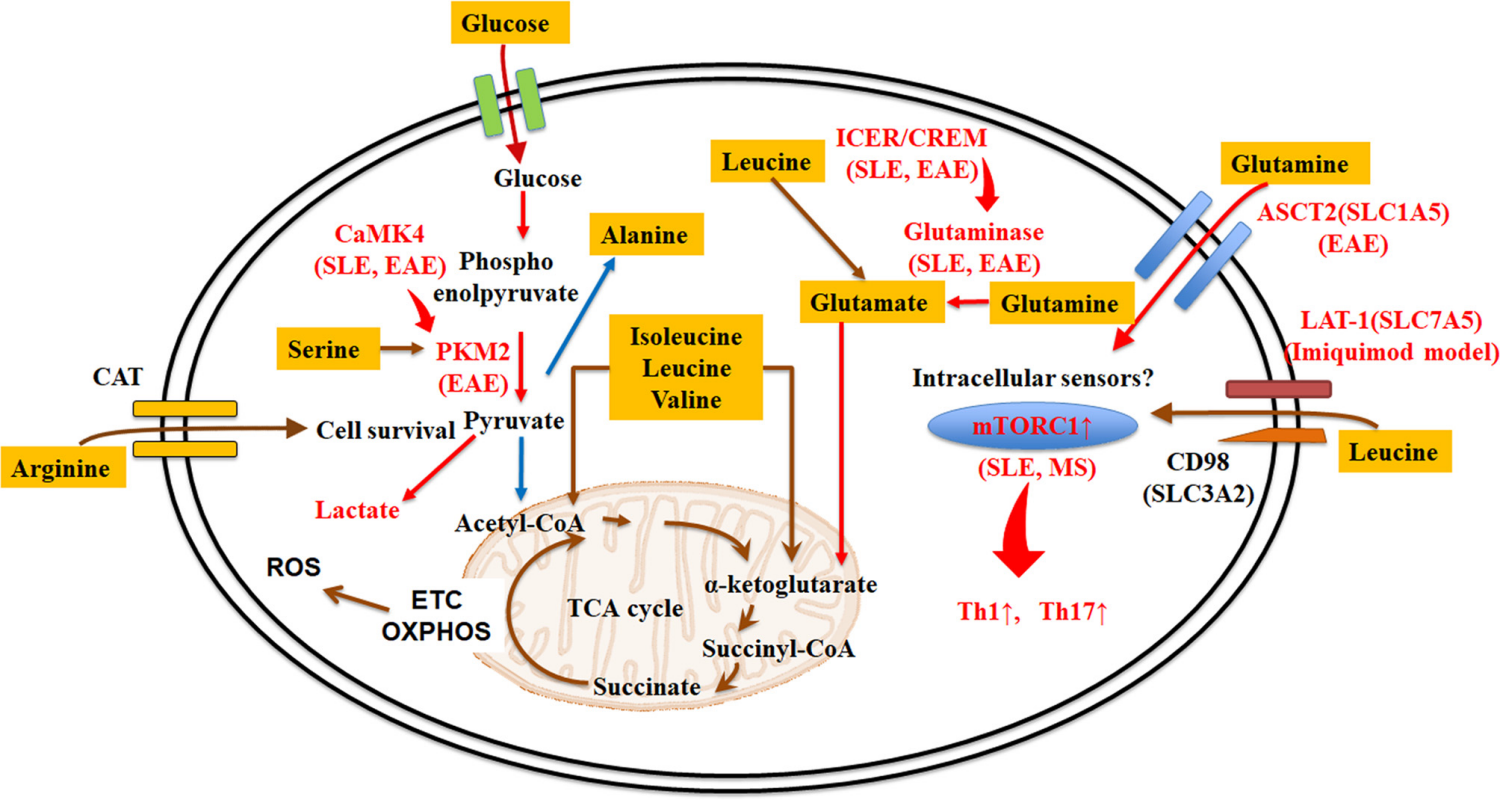
Amino acid transporters and metabolism in lupus T cells. Amino acid acquisition is crucial for cell function. Amino acid transporters play central roles in acquiring amino acids from the external environment. Some amino acids (e.g. leucine, methionine, glutamine, arginine, and alanine) are more essential than other amino acids in during T cell activation and expansion, or in determining different T cell fates in autoimmune diseases. Red arrows or letters indicate “enhance or active”, whereas blue arrows indicate “inhibit or inactivate”. ASCT2, alanine-serine-cysteine transporter 2; CaMK4, calcium/calmodulin–dependent protein kinase IV; CAT, cationic amino acid transporters; CREM, cAMP response element modulator; EAE, experimental autoimmune encephalomyelitis; ETC, electron transport chain; ICER, inducible cAMP early repressor; LAT-1, large neutral amino acid transporter 1; mTORC, mammalian target of rapamycin complex; OXPHOS, oxidative phosphorylation; PKM2, pyruvate kinase muscle isozyme 2; ROS, reactive oxygen species; SLE, systemic lupus erythematosus; TCA cycle, tricarboxylic acid cycle.

Amino acid transporters are important in transporting amino acids from the environment into the cell (19). T-cell receptor (TCR) stimulation triggers dramatic metabolic changes including increased glycolysis, pentose phosphate pathway activity, and glutaminolysis (19, 20). SLC7A5, known as large neutral amino acid transporter 1 (LAT-1), is a transporter dedicated to the transport of essential amino acids (21). SLC3A2, also known as CD98, is a transmembrane protein, which chaperones amino-acid transporters, including SLC7A5 SLC7A6, SLC7A7, SLC7A8, SLC7A10, and SLC7A11 (12), and enables them to execute their function. The LAT-1/CD98 heterodimer transports large hydrophobic amino acids, including the seven essential amino acids leucine, isoleucine, histidine, valine, phenylalanine, tryptophan, and methionine. Notably, the expression of LAT-1 and CD98 in T cells is induced after activation (19). *Slc7a5^-/-^* CD4^+^ T cells cannot respond to antigen, undergo clonal expansion or effector cell differentiation. Although *Slc7a5^-/-^* CD4^+^ T cells do not differentiate into Th1 and Th17 cells, differentiation into iTreg is not affected (22). LAT-1 deletion or inhibition blocks the expansion of IL-17 secreting γδ and CD4^+^ T cells in both human cells and imiquimod (a TLR7 agonist)-induced lupus and psoriasis-like animal models (**Figure 1**). The heterodimer comprising CD98 and SLC7A7 transports among other amino acids lysine, arginine, methionine, leucine, alanine, and cysteine (12). Interestingly, whole-exome sequencing in patients with childhood-onset SLE identified a *SLC7A7* mutation to be linked to disease expression (23).

Alanine is also important in T cell activation. It is transported through SLC38A1 in CD4^+^ T cells and TCR stimulation induces its expression (12). Alanine deprivation impairs naïve and memory T cell activations, but it does not affect T cell effector functions (24). Although alanine can be made from pyruvate by a single transamination, extracellular alanine is used mainly for protein synthesis (12, 24).

Glutamine is the most abundant amino acid in the serum (25, 26). T cell stimulation promotes a rapid increase of glutamine uptake and activated T cells need more glutamine than naïve T cells (27). SLC1A5, known as alanine-serine-cysteine transporter 2 (ASCT2), is a transporter of neutral amino acids including glutamine (28). Although *Slc1a5^-/-^* CD4^+^ T cells do not affect TCR-mediated activation, deletion of *Slc1a5* impaired Th1 and Th17 cell differentiation (**Figure 1**) (27).

Arginine is transported through cationic amino acid transporters (CAT) (29), which are shared by lysine and ornithine. Elevation of arginine levels induces metabolic changes including a shift from glycolysis to oxidative phosphorylation in activated T cells and promotes the generation of central memory-like cells (30). Arginine and the transporter CAT-1 (SLC7A1) are also requisite for human T cell survival (31).

These findings demonstrate distinct roles for amino acid transporters in TCR/CD3-mediated T cell stimulation, differentiation, and function and indicate that manipulation of these transporters could serve therapeutic approaches for autoimmune diseases including SLE (**Figure 1**). Because several other amino acid transporters have not been studied carefully in T cells, further research is needed.

## Amino Acid Sensors

Although multiple mechanisms are involved in sensing amino acids within the intracellular space, it has been well established that the presence or absence of amino acids is sensed by distinct signaling pathways which involve the mechanistic target of rapamycin (mTOR) or the general control nonderepressible 2 (GCN2) (32, 33).

mTOR activity is regulated by amino acid availability, energy levels, and growth factors (34). In mammalian cells mTOR forms two distinct complexes: the mTORC complex 1 (mTORC1) and mTORC2. In fact, mTORC1 senses various stress signals, including the accumulation of amino acids such as leucine, isoleucine, kynurenine, and glutamine (35, 36). Glutamine activates mTORC1 *via* its metabolic product α-ketoglutarate which is generated during glutaminolysis (37). Inhibition of the first enzyme of glutaminolysis, glutaminase 1, reduces the activity of mTORC1 under Th17-polarized conditions (38). mTORC activity is enhanced in Th17 cells and IL-4-producing double negative T cells resulting in the proinflammatory profile recorded in patients with SLE (39). During Th17 cell differentiation, mTOR is required for the induction of hypoxia-inducible factor 1α (HIF1α) which enhances glycolysis (40). In Th1 and Th17 cells, mTORC1 activity, and glycolysis are increased compared with Tregs and Tfh cells (40, 41). Sirolimus, a mTOR inhibitor, was reported to improve disease activity in patients with refractory SLE in a single-arm, open-label, phase I/II trial (42), and other non-randomized controlled studies have reported that sirolimus is efficacious in patients with SLE (**Table 1**) (43). Sirolimus normalized Th17/Treg balance and TCR-induced Ca^2+^ fluxing in patients with SLE (44, 45). Besides the effect on T cells, inhibition of mTOR in plasmacytoid dendritic cells reduced the production of type I interferons (58) and B cell stimulating factor BAFF-mediated B cell activation (59, 60). These results indicate that sirolimus can modify T, B, and plasmacytoid dendritic cell function (46). Further randomized controlled trials are needed to prove the efficacy and record the side effects of sirolimus in patients with SLE (47).

**Table 1:** Tentative therapeutic targets identified in studies of amino acid metabolism in T cells.

Therapeutic target	Therapy	Effect on T cells	Effects on lupus	References
**Amino acid transporters**				
LAT-1(SLC7A5)/CD98(SLC3A2)	JPH203	Cannot respond to antigen, undergo clonal expansion or effector differentiation	Unknown	(12, 19, 21, 22)
(Transporter for Leu, Ile, His, Val, Phe, Trp, Met, and Tyr)		Reduces Th1 and Th17 cell differentiation		
ASCT2(SLC1A5)	V-9302	Reduces Th1 and Th17 cell differentiation	Unknown	(27, 28)
(Transporter for Gln, Ala, Ser, Cys, Asp, and Thy)	GPNA			
CAT-1 (SLC7A1)	NEM	Requisite for T cell survival	Unknown	(29, 30)
(Transporter for Arg, Lys, and Orn)				
**Amino acid sensors**				
mTOR signaling	Sirolimus*	Inhibits Th17 cell differentiation	Reduces disease activity (mouse and human)	(39–48)
		Promotes Treg cell differentiation		
**Amino acid metabolism**				
** Glutamine metabolism**				
Glutaminase 1	BPTES		Reduces disease activity	(38, 49, 50)
	CB-839, 968	Reduces Th17 cell differentiation	Improve kidney disease (mouse)	
GOT1	AOA	Reduces Th17 cell differentiation	Unknown	(51)
Glutaminolysis	DON	Reduces the frequency of Tfh cells	Reduces dsDNA antibody production (mouse)	(52)
** Cysteine metabolism**	NAC*	Inhibits mTOR activity	Reduces disease activity	(53–57)
			Improve kidney disease (mouse and human)	

LAT-1, large neutral amino acid transporter 1; ASCT2, alanine-serine-cysteine transporter 2; CAT, cationic amino acid transporters; mTORC, mammalian target of rapamycin complex; GPNA, L-γ-glutamyl-p-nitroanilide; NEM, N-ethylmaleimide; GOT-1, glutamate oxaloacetate transaminase 1; AOA, (aminooxy)acetic acid; DON, 6-Diazo-5-oxo-L-norleucine; NAC, N- acetyl cysteine; Leu, leucine; Ile, isoleucine; His, histidine; Val, valine; Phe, phenylalanine; Trp, tryptophan; Met, methionine; Tyr, tyrosine, Gln, glutamine; Ala, alanine, Ser, serine; Cys, cysteine; Asp, asparagine, Thr, threonine; Arg, arginine Lys; lysine, Orn, Ornithine. *; Clinical trials of these therapies are ongoing.

GCN2, a serine/threonine-protein kinase, also senses amino acid starvation by detecting uncharged transfer RNA (33, 61). It plays a vital role in the control of amino acid metabolism as a response to nutrient deprivation. *Gcn2* deficiency significantly inhibits *in vitro* differentiation of Th9 cells but not Th1, Th2, and Treg cells in mouse model, and it ameliorated allergic airway inflammation in mice (62). On the other hand, myeloid cell deletion of *Gcn2* in lupus-prone mice resulted in increased immune cell activation, humoral autoimmunity, renal pathology, and mortality (63). These results suggest that therapeutic inhibition of GCN2 should not be considered to treat SLE.

## Glutamine Metabolism

Glutaminolysis has a vital role in energy production in proliferating cells including T cells. Because of the indispensable roles of glutaminolysis in the generation of pro-inflammatory effector T cells Th1 and Th17 cells, enzymes involved in glutaminolysis have been studied extensively.

Glutaminase, in charge of converting glutamine to glutamate, promotes Th17 cells through distinct mechanisms (38, 49). Glutaminase expression is controlled by the transcription factor inducible cAMP early repressor (ICER)/cAMP response element modulator (CREM) (38), which is known to be overexpressed in T cells both from patients with SLE or MRL/*lpr* lupus-prone mice (64, 65). The glutaminase 1 inhibitor Bis-2-(5-phenylacetamido-1,3,4-thiadiazol-2-yl)ethyl sulfide (BPTES) reduces Th17 cell differentiation and disease activity in animals subjected to experimental autoimmune encephalomyelitis (EAE) (38). BPTES also ameliorates disease activity in MRL/*lpr* mice (50). Glutamate oxaloacetate transaminase 1 (GOT1), which converts glutamate to α-ketoglutarate, an intermediate of the TCA cycle, also contributes to enhance Th17 cell differentiation through epigenetic processes (51). Selective inhibition of GOT1 with aminooxy acetic acid (AOA) treatment or short hairpin RNA (shRNA) silencing markedly decreased Th17 differentiation of murine T cells (51). Systemic AOA treatment or adoptive transfer of *Got1* knockdown Th17-polarized T cells ameliorated EAE (51). Furthermore, inhibition of glutaminolysis with the glutamine analog 6-Diazo-5-oxo-L-norleucine (DON) reduces the frequency of Tfh cells, exogenous antigen-specific germinal center responses, and the production of dsDNA antibody in lupus-prone B6.*Sle1.Sle2.Sle3* mice after T cell-dependent immunization (52).

## Branched-Chain Amino Acid Metabolism

The branched-chain amino acids (BCAAs) include leucine, isoleucine, and valine. As the most abundant of essential amino acids, BCAAs are not only the substrates for synthesis of nitrogenous compounds, but they also serve as signaling molecules regulating the metabolism of glucose, lipid, and protein synthesis, intestinal health, and immunity through special signaling networks, especially the phosphoinositide 3-kinase/protein kinase B/mTOR (PI3K/AKT/mTOR) signal pathway. The leucine antagonist *N*-acetyl-leucine amide (NALA) inhibits mTORC1 activity and T cells function, impairs IL-2 and IFNγ production in *in vitro* Th1 polarized murine T cells (66). Leucine is also essential for Treg cell function. Leucine promotes mTORC1 activity in Treg cells *via* the small G proteins RagA/B and Rheb1/2 to drive their suppressive activity by inducing the expression of inducible T cell costimulator (ICOS) and CTLA4. Mice bearing RagA-RagB- or Rheb1-Rheb2-deficient Treg cells developed a *Scurfy*-like autoimmune disease and have reduced effector Treg cell accumulation and function (48).

Unlike most other essential amino acids, BCAAs catabolism is initially catalyzed either by transamination by branched-chain amino acid aminotransferases (BCAT) or decarboxylation by branched-chain α-keto acid dehydrogenase enzyme complex (BCKDC). After these reactions BCAA metabolites are further converted to acetyl-CoA and succinyl-CoA and participate in the TCA cycle (67). In CD4^+^ T cells, BCAT negatively regulates mTOR and glycolysis. Activated T cells from cytosolic branched chain aminotransferase (BCATc)-deficient mice show increased mTORC1 activation compared to T cells from control mice. Furthermore, T cells from *Bcatc^-/-^* mice display higher rates of glycolysis (68). In another study, the oral administration of a leucine analogue, ERG240, selectively inhibited the activity of BCAT1, reduced the severity of collagen-induced arthritis in mice, and crescentic glomerulonephritis in rats (69).

## Serine Metabolism

Serine is used in proliferating cells for protein synthesis as well as the synthesis of other amino acids, such as glycine and cysteine (70). Serine-derived glycine is used in nucleotide synthesis. Moreover, serine is also a precursor for the synthesis of lipids, such as phosphatidylserine and sphingolipids, which have central roles in apoptotic cell clearance and immune cell activation, respectively (71, 72). A key molecule which is associated with serine is the M2 isoform of pyruvate kinase (PKM2) because it ligates and allosterically activates its activity (73). Even in the absence of exogenous serine, PKM2 expression contributes to endogenous serine synthesis and to the maintenance of mTORC1 activity (74).

Upon T cell activation, upregulated enzymes of the serine, glycine, one-carbon (SGOC) metabolic network, increase processing of serine into one-carbon metabolism. Extracellular serine is required for optimal T cell proliferation both *in vitro* and *in vivo.* Shortage of dietary serine impairs pathogen-driven expansion of T cells *in vivo*. Serine supplies glycine and one-carbon units for *de novo* nucleotide biosynthesis in proliferating T cells, and one-carbon units from formate can rescue T cells from serine deprivation (75).

We previously reported that calcium/calmodulin–dependent protein kinase IV (CaMK4) binds to PKM2 and promotes pyruvate kinase activity. Activated PKM2 is requisite for the Th1 and Th17 differentiation (76). Because inhibition of CaMK4 ameliorates pathogenesis of SLE though a Th17 cell manner (77, 78), the serine/PKM2 metabolism axis represents a hub of abnormal T cells in autoimmunity and needs further attention.

Serine also supports mitochondrial metabolism. In Jurkat cells, the catabolic enzyme serine hydroxymethyltransferase (SHMT2) is required for mitochondrial and respiratory activity (79). It has been also shown that SHMT2 promotes inflammatory cytokine signaling, including that of type I interferons, by interacting with the deubiquitylating BRCC36 isopeptidase complex (BRISC) (80). Since it has been recently shown that an inactive form of SHMT2 dimer has the capacity to bind and inhibit BRISC (80), control of the SHMT2-BRISC interaction may represent a new target to control autoimmune diseases.

## Glutathione/Cysteine Metabolism

Glutathione is made from three amino acids: cysteine, glutamate, and glycine. Glutathione is important in the antioxidant defense, nutrient metabolism, and regulation of cellular events including gene expression, DNA and protein synthesis, cell proliferation and apoptosis, signal transduction, cytokine production and protein glutathionylation (81). Glutathione reduces intracellular reactive oxygen species (ROS) levels and inhibits Th17 cell differentiation (49, 82). Glutathione is reported to be decreased in the peripheral blood of patients with SLE (83). Glutathione regulates the elevation of mitochondrial transmembrane potential, which in turn activates mTOR in T cells from patients with SLE (53, 84). To date, N-acetylcysteine (NAC) has been used to correct glutathione levels because NAC is the cell-permeable precursor of cysteine which is the rate-limiting constituent of *de novo* reduced glutathione (53–55). Administration of NAC improves lupus disease activity and ameliorates organ damage mainly by blocking the mTOR pathway in T cells in humans and mice with SLE (56, 57).

Because cysteine contains sulfur, cysteine supports sulfur-dependent metabolism. As discussed above, cysteine is a key amino acid for glutathione function, as it supplies the sulfur necessary for the formation of the disulfide bridge in the glutathione disulfide (13), but its roles extend beyond glutathione synthesis. In humans, naïve T cells express none or very low levels of cystine and cysteine transporters. Thus, early T cells activation does not require cystine and cysteine. However, upon activation, T cells rapidly upregulate the expression of cystine and cysteine transporters and display dependency on exogenous supply of cystine/cysteine for their proliferation (85).

## Metabolism of Other Amino Acids

Tryptophan, an essential amino acid used for the biosynthesis of crucial compounds, including 5-hydroxytryptamine (5-HT, serotonin) and kynurenine, is important in T cell function. Indoleamine-2,3-dioxygenase 1 (IDO-1) catabolizes tryptophan to kynurenine and T cells require tryptophan for proliferation and activation (13). Accordingly, IDO-1 inhibits T cell activation and Treg cell differentiation of human and murine T cells (86–89). The dysbiotic gut microbiota of lupus-prone mice which is characterized by altered distribution of tryptophan metabolites in the feces of the mice, including an increase in kynurenine levels, has been linked to the production of autoantibodies and autoimmune pathology (90). Low dietary tryptophan prevents disease activity of the lupus-prone mice, whereas high dietary tryptophan has the opposite effect (90).

Methionine can affect the epigenetic reprogramming in CD4^+^ T cells (91). Activated T cells transport methionine *via* SLC7A5 (92). Methionine serves as the major substrate for the biosynthesis of S-adenosyl-L-methionine (SAM) (91, 93). SAM functions as a substrate for epigenetic modifications. Methionine restriction reduces histone H3K4 methylation at promoter regions of genes associated with Th17 cell proliferation and cytokine production in murine T cells (91).

## Conclusions

During the last decade great progress has been achieved in the field of immunometabolism. It has now been established that T cell metabolism controls the fate and function of T cells. Amino acids are also crucial in T cell survival, function and differentiation. Besides glycolysis, amino acid metabolism is also involved in the pathogenesis in SLE and by inference to other autoimmune diseases. Although 2-deoxy-d-glucose monotherapy has partial efficacy in improving disease in lupus-prone mice, when combined with metformin, a mitochondrial electron transport chain complex I inhibitor, it leads to normalization of T cell metabolism and reversal of disease activity (94). These results revealed that monotherapy targeting only glycolysis is not sufficient to treat lupus-prone mice. Thus, the focus of research on T cell metabolism in lupus is expanding our understanding of amino acid metabolism.

Although many reports have shown that some metabolic pathways involving amino acids including glutamine, tryptophan, and cysteine can serve as therapeutic targets in lupus-prone mice, the tentative therapeutic targeting of metabolic pathways of other amino acids remains unclear. Sirolimus and NAC are undergoing rigorous clinical trials in patients with SLE (42, 43, 80) and they may end up serving as significant entries in the list of available therapeutic tools for these patients. There are though several challenges to overcome in order to exploit additional amino acid-related treatment targets. Although many studies using mouse models have revealed potential therapeutic targets in amino acid metabolism, further insights are needed from the *ex vivo* study of immune cells from patients with SLE. Such studies should be followed by properly designed clinical trials in patients with SLE and probably other autoimmune diseases. As all drugs display invariably side effects, cell/tissue targeted delivery should be considered (73, 95, 96).

In this brief review we presented evidence that amino acids are important in T cell function and aberrant metabolism may be linked to autoimmunity and related pathology. It appears that their central role in the control of the immune response is underwritten by being indispensable for the generation of building blocks needed for cell proliferation, the generation of energy by controlling metabolic pathways, the control of epigenetic pathways, the production of phospholipids and the control of oxidative stress.

Amino acids and products of metabolic processes dictate the effector function of T cells and determine whether they will serve as regulators, instigators of inflammation or effectors of cytotoxicity. Alterations of the levels of metabolites within immune cells can be achieved by simply changing their levels in the environment or modulating the activity of transporters and intracellular metabolic enzymes. Drugs altering metabolism or supplementation of amino acids or metabolites or their precursors may prove of great value as modulators of T cell functions in the treatment and well-being of patients with autoimmune disease.

## Author Contributions

MK, NY, and GT conceptualized the article, reviewed the literature, and wrote the manuscript. All authors contributed to the article and approved the submitted version.

## Funding

This work was supported by the United States National Institutes of Health grants R01AR064350, R37 AI 49954 (to GT), AMED under Grant Number JP 20ek0410078, a SENSHIN Medical Research Foundation grant (to MK), and Gilead Sciences research scholars program in rheumatology (to NY).

## Conflict of Interest

MK reports grants from GlaxoSmithKline plc, Mitsubishi Tanabe, Astellas, Sanofi, Taisho Pharmaceutical, NIPPON SHINYAKU CO., LTD., and Taiju Life Social Welfare Foundation, outside the submitted work. GT reports consultancies, speaking fees, or honoraria from Janssen, Novartis, ABPRO, Silicon Therapeutics, A2 Thera.

The remaining author declares that the research was conducted in the absence of any commercial or financial relationships that could be construed as a potential conflict of interest.

The handling editor declared a past collaboration with the authors GT and NY.
